# A techno-economic perspective on efficient hybrid renewable energy solutions in Douala, Cameroon’s grid-connected systems

**DOI:** 10.1038/s41598-024-64427-4

**Published:** 2024-06-12

**Authors:** Reagan Jean Jacques Molu, Serge Raoul Dzonde Naoussi, Mohit Bajaj, Patrice Wira, Wulfran Fendzi Mbasso, Barun K. Das, Milkias Berhanu Tuka, Arvind R. Singh

**Affiliations:** 1 Technology and Applied Sciences Laboratory, U.I.T. of Douala, University of Douala, P.O. Box 8689 Douala, Cameroon; 2 Department of Electrical Engineering, Graphic Era (Deemed to be University), Dehradun, 248002 India; 3 Hourani Center for Applied Scientific Research, Al-Ahliyya Amman University, Amman, Jordan; 4 Graphic Era Hill University, Dehradun, 248002 India; 5 Laboratoire IRIMAS, University de Haute Alsace, 61 Rue Albert Camus, 68200 Mulhouse, France; 6 School of Engineering, Edith Cowan University, Joondalup, WA 6027 Australia; 7 Department of Electrical and Computer Engineering, College of Engineering, Addis Ababa Science and Technology University, Addis Ababa, Ethiopia; 8 Department of Electrical Engineering, School of Physics and Electronic Engineering, Hanjiang Normal University, Hubei Shiyan, 442000 P. R. China

**Keywords:** Optimization, Techno-economic, Hybrid renewable energy, Grid-connected, Energy science and technology, Engineering, Mathematics and computing

## Abstract

Cameroon is currently grappling with a significant energy crisis, which is adversely affecting its economy due to cost, reliability, and availability constraints within the power infrastructure. While electrochemical storage presents a potential remedy, its implementation faces hurdles like high costs and technical limitations. Conversely, generator-based systems, although a viable alternative, bring their own set of issues such as noise pollution and demanding maintenance requirements. This paper meticulously assesses a novel hybrid energy system specifically engineered to meet the diverse energy needs of Douala, Cameroon. By employing advanced simulation techniques, especially the Hybrid Optimization Model for Electric Renewable (HOMER) Pro program, the study carefully examines the intricacies of load demands across distinct consumer categories while accommodating varied pricing models. The paper offers a detailed analysis of the proposed grid-connected PV/Diesel/Generator system, aiming to gauge its performance, economic feasibility, and reliability in ensuring uninterrupted energy supply. Notably, the study unveils significant potential for cost reduction per kilowatt-hour, indicating promising updated rates of $0.07/kW, $0.08/kW, and $0.06/kW for low, medium, and high usage groups, respectively. Furthermore, the research underscores the importance of overcoming operational challenges and constraints such as temperature fluctuations, equipment costs, and regulatory compliance. It also acknowledges the impact of operational nuances like maintenance and grid integration on system efficiency. As the world progresses towards renewable energy adoption and hybrid systems, this investigation lays a strong foundation for future advancements in renewable energy integration and energy management strategies. It strives to create a sustainable energy ecosystem in Cameroon and beyond, where hybrid energy systems play a pivotal role in mitigating power deficiencies and supporting sustainable development.

## Introduction

### Background of study

**Figure 1:**
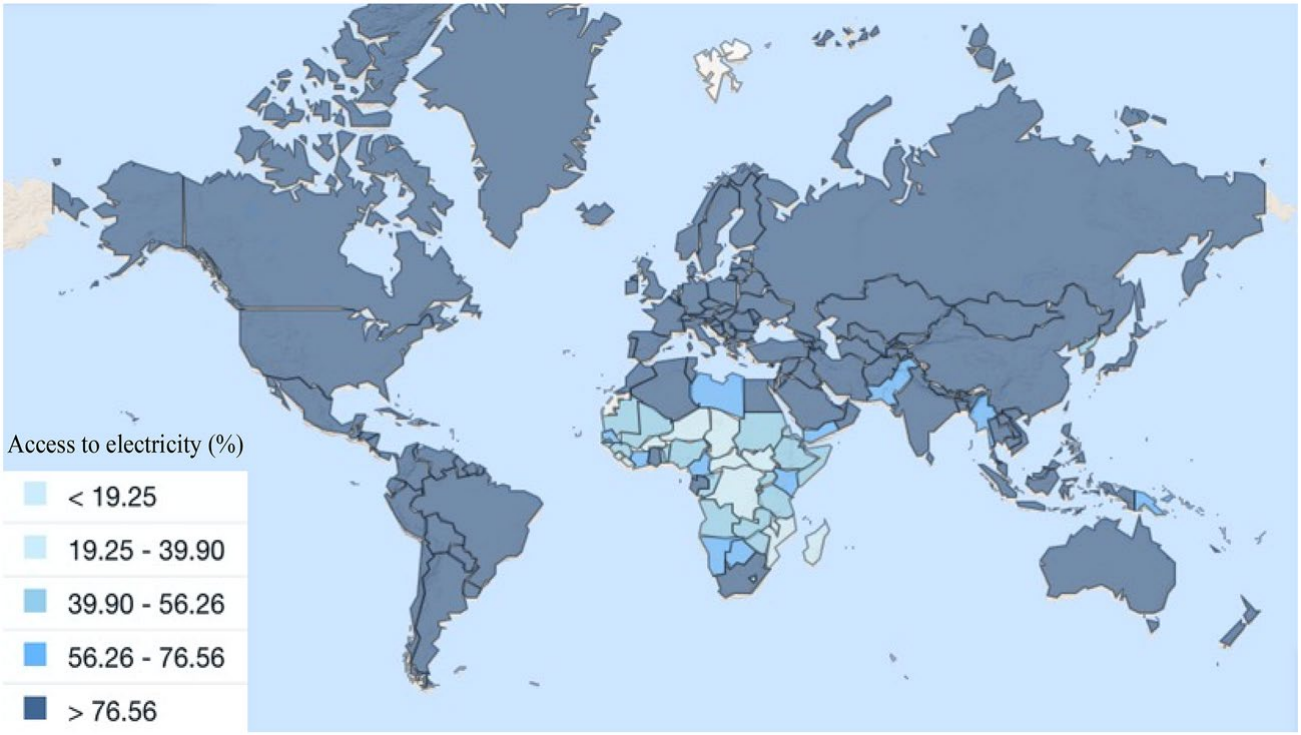
World population with access to electricity by country in 2020^6^.

Electricity access is crucial for a country's prosperity, affecting well-being, economic progress, and public health^1^. However, it remains limited in many parts of Africa and the world, with an estimated one billion people facing challenges with inconsistent access to energy by 2020^2^. Sub-Saharan Africa has around 100 million people living with limited or no access to power (Fig. 1). To address this issue, researchers are increasingly focusing on renewable energy sources, particularly solar and wind power^3^. Sustainable Development Goal 7 emphasizes the importance of affordable, reliable, and sustainable energy^4^. Cameroon has a diverse array of alternative energy sources, but their efficacy varies across locations. Hybridization presents a promising approach to optimize effectiveness by harmonizing diverse renewable energy sources, offering enduring fiscal, environmental, and ecological benefits^5^. Decentralized energy systems are emerging as a compelling solution to inadequate electricity access in sub-Saharan Africa, offering environmental friendliness, reduced operational expenses, and the capacity to leverage clean energy technologies.

### Literature survey

Due to the seasonal variations in solar radiation and wind speed, the dependability of renewable energy sources such as solar and wind experiences notable fluctuations^7^. A remedy employed to tackle this challenge is the integration of Hybrid Renewable Energy Systems (HRES). By capitalizing on optimal hours and seasons for energy generation, HRES mitigates reliability concerns and presents cost reductions compared to relying solely on a single renewable energy source^8^. However, establishing the ideal system size to fulfill specific load demands at a given site remains intricate due to the unpredictable nature of energy sources, intricacies in efficient cost modeling, and the time-intensive nature of optimization algorithms inherent to the context of optimal design^57,58^.

To surmount this challenge, software solutions are imperative, and the literature highlights various software packages that streamline the optimization process^9^. Among them, the Hybrid Optimization Model for Electric Renewable (HOMER), developed by the National Renewable Energy Laboratory (NREL), stands out as a popular choice. It allows for the streamlined and accurate evaluation of diverse combinations, facilitating the intricate optimization process^10^. To glean insights from previous work in this domain, a comprehensive literature review was undertaken. Rosales-Perez et al.^11^ investigated integrating two solar thermal collector technologies to improve the economic viability of solar systems in district heating applications. They evaluated the energy and financial possibilities of hybrid systems that combine flat plate and parabolic trough collectors at different industrial process temperatures and radiation levels. The hybrid system attained significant solar percentages while maintaining a reduced levelized cost of heat in comparison to solo parabolic trough collector systems with smaller solar field sizes. In another study situated in Newfoundland, Canada, Khan et al.^12^ embarked on a research project that delved into the viability of harnessing hydrogen as a power source within a combined energy system. The study employed the HOMER software to evaluate diverse renewable and conventional energy alternatives, along with various energy storage methods, via simulation and optimization techniques. The study's outcomes indicated the potential feasibility of a wind-diesel storage system, considering prevailing cost dynamics. However, an interesting proposition emerged, suggesting that a wind-fuel cell system could become more attractive if there was an approximate 15% reduction in fuel cell costs.

Indonesian researchers undertook additional research aimed at establishing an autonomous power system to fulfill the energy requirements for administrative and communal functions across three distinct regions within Maluku, Indonesia. In this context, the work conducted by Putra et al.^13^ concentrated on the examination of three specific locations: Wairtan, Klishatu, and Leiting Village. Employing software such as HOMER Pro, PVsys, and PVsol, the study analyzed the influence of administrative and communal energy demands. The results from this investigation could be harnessed to enhance both the rural electrification initiative and the Bright Indonesia campaign, as proposed by the authors.

Gomez et al.^14^ created an optimization model to handle operational revenue in liberalized power markets, focusing on the Italian and Iberian day-ahead power markets. The study revealed that the annual net income of the hybrid PV-wind-storage power plant may improve by 4% when compared to operating each component separately. Hussam et al.^15^ performed research on the possibility for hydrogen generation via a hybrid energy system at the Shagaya renewable power plant. Techno-economic and optimization evaluations were used to identify the most cost-effective designs that would enhance the renewable portion and decrease greenhouse gas emissions. The improved system produced 111,877 kg of green hydrogen annually and reduced carbon dioxide emissions by 14,819 kg annually. The sensitivity study revealed that the cost of energy (COE) is more sensitive to changes in PV prices compared to wind turbines and electrolyzers. Assessing the techno-economic viability of hybrid energy systems (HES) that combine Photovoltaic (PV) and Reformer Fuel-Cell (RF-FC) components and are connected to the grid, Dekkiche et al.^16^ conducted a study employing diverse solar PV tracking methodologies. The evaluation involved the utilization of HOMER Pro software to simulate and evaluate the feasibility and expenses associated with different system configurations throughout their operational lifespans. The optimal financial outcomes appear achievable through skillful engineering of a grid-connected hybrid PV/RF-FC energy system, particularly employing a Vertical Single Axis Tracker (VSAT).

Salem et al.^17^ suggested a polygeneration system that uses renewable energy to create several energy forms and includes storage to reduce the effects of unpredictable weather. The research used transient simulation software TRNSYS® to dynamically analyze, model, optimize, and simulate the system under Gujrat, Pakistan weather conditions. The system facilitates several applications such as air-conditioning, heating, electric power supply for electric cars, building load & national grid, hydrogen for internal combustion engines, fuel cell electric vehicles, and industrial usage. In a distinct investigation carried out by Chisale et al.^18^, six diverse hybrid system scenarios were scrutinized, all geared towards achieving heightened electricity reliability, diminished grid reliance, and reduced costs for a school setting. The optimization of system configuration was determined using HOMER Pro software, while subsequent analysis was conducted employing CRITIC-PROMETHEE II methodologies. The most advantageous configuration consisted of an amalgamation of grid electricity, solar photovoltaic (PV), and biogas-generated power. The calculated levelized cost of electricity for this proposed system stands at 0.095 $/kWh, signifying a cost advantage compared to the prevailing cost of Malawi's grid electricity, which stands at 0.11 $/kWh. The study recommends that educational institutions and governmental entities allocate resources towards the advancement of alternative energy sources as a strategy to mitigate greenhouse gas emissions.

The city of Duqm is positioned in Oman's Al Wasta Governorate and relies on a power supply mix consisting of 10 diesel generators, collectively capable of producing 76 MW, along with supplementary rental power sources delivering 18 MW. Al-Badi et al.^19^ introduce an innovative strategy that advocates for a complete shift in the city's electric power sourcing from diesel to hydrogen, thereby embracing renewable sources. The assessment of the microgrid's technical and financial performance was conducted using the HOMER Pro software. The outcomes highlight that a combination of solar, wind, and fuel cell technologies represents the most practical and cost-effective approach. Notably, when compared to batteries, hydrogen emerges as a more economically feasible option for extended energy storage requirements.

Pires et al.^20^ introduced a multi-objective optimization approach for combining wind and solar energy production with battery energy storage, focusing on tariff policy challenges in residential settings linked to the grid. The Response Surface Methodology (RSM) was used to create models for environmental and financial functions, which were then implemented in Brazilian towns. The analysis determined that areas with conducive environmental conditions and elevated energy prices were economically feasible, with NPV values ranging from R$ − 76,080.94 to R$ 69,675.23. Solar PV technology, although cost-effective, necessitated more storage capacity, leading to a preference for setups with higher wind output.

In their investigation, He et al.^21^ identified the most effective combination of renewable energy resources capable of both mitigating CO_2_ emissions and ensuring a reliable electricity supply. The key influential factors include constraints imposed by land availability and penalties associated with carbon dioxide emissions. The optimization results unveiled a net present cost (NPC) of $1.02 million and a levelized cost of electricity (COE) amounting to 0.188 $/kWh. To ensure broader applicability, the study underwent sensitivity analyses.

Youssef et al.^22^ developed a cost-effective renewable energy system utilizing the HOMER program to reduce energy expenses and generate power efficiently. They used data from NASA and real-time field data on wind and solar resources to compare lithium-ion and lead acid batteries and identify the most cost-effective choice. The hybrid power system combines PV, wind, and biomass generators to fulfill energy needs, using excess energy to recharge battery banks when output is low. Syahputra et al.^23^ directed their efforts towards establishing a grid-connected hybrid system for Yogya-karta that harnesses the Java-Madura-Bali electricity network. The system's hydro and solar potential for electricity generation had yet to be fully optimized. The PSO algorithm was employed to determine the system's capacity, while accounting for factors such as capital costs, sell-back pricing, COE, and NPC. The study identified peak load periods from 6 p.m. to 9 p.m. and off-peak hours from 12 p.m. to 4 a.m.

Molu et al.^24^ conducted a study on the lack of accessible and reliable electricity in Cameroon, identifying it as a hindrance to the nation's progress. They explored the feasibility of implementing Hybrid Renewable Energy Systems (HRES) to meet the energy demands of three small communities on Manoka Island, Douala, Cameroon. Through technical, environmental, and economic analysis, they assessed the potential of integrating solar panels, wind turbines, battery cells, fuel cell generators, biogas, and an electrolyzer in an off-grid HRES system. The study demonstrated low unit energy costs and a compelling net present value, highlighting the cost-effectiveness of the arrangement. In essence, their research represents a pioneering case study in sustainable electricity provision, contributing significantly to knowledge on renewable energy and its potential for sustainable development and energy security in Cameroon.

In another study by Das et al.^25^, the feasibility of integrating three distinct electrochemical energy storage technologies-lead acid, lithium-ion, and vanadium redox flow-into independent hybrid energy systems was examined. The findings indicated that utilizing a hybrid system with a cyclic charging strategy led to decreased energy costs, albeit with slightly higher lifecycle emissions compared to load-following strategies. Hybrid alternatives employing vanadium redox flow technology presented the most economically efficient energy solution, with costs ranging from 0.126 to 0.187 dollars per kilowatt-hour. These options also demonstrated favorable environmental impacts throughout their lifespan, with emissions ranging from 46,258 to 104,664 kg of CO_2_-equivalent per year. Sensitivity analysis results indicated that decreased energy costs corresponded to reduced reliability and lifecycle emissions. This study offers crucial insights for energy planners in selecting optimal battery technology and dispatch strategies that yield superior outcomes across technical, economic, environmental, and social dimensions.

### Research gaps and study contributions

Limited research has investigated the utilization of renewable energy-derived electricity for households in alignment with grid tariffs, as indicated by the existing literature and research deficiencies. Additionally, there is a scarcity of studies appraising the feasibility of on-grid renewable energy sources in varied global locations. Our research seeks to address several critical gaps in the current understanding of hybrid renewable energy solutions in the context of Douala's grid-connected systems. Specifically, the authors identify the following research gaps:

- Existing studies often lack comprehensive techno-economic analysis of hybrid renewable energy systems tailored to the specific socio-economic and environmental conditions of Douala.
- The perspectives of key stakeholders, including policymakers, energy providers, and local communities, are often overlooked in the design and implementation of renewable energy projects in Douala.
- There is a scarcity of in-depth case studies that examine the feasibility and viability of hybrid renewable energy solutions within the unique regulatory and infrastructural framework of Douala.

This study aims to address several critical gaps in the understanding and implementation of hybrid renewable energy solutions in Douala's grid-connected systems. The key contributions of this research are as follows:

- Conducted a detailed techno-economic analysis of hybrid renewable energy systems in Douala, incorporating capital costs, operational expenses, and environmental impacts.
- Actively involved key stakeholders, including policymakers, energy providers, and local communities, to enhance the practical relevance of the findings.
- Presented detailed case studies of hybrid renewable energy projects in Douala, highlighting technological innovations, regulatory frameworks, and socio-economic impacts.
- Determined optimal configurations of hybrid renewable energy systems based on residential energy demand patterns and solar PV potential in Douala, evaluating efficiency using metrics like Net Present Cost (NPC) and Cost of Energy (COE).
- Addressed gaps in existing literature by providing a comprehensive analysis of consumption billing and energy dynamics in Douala's grid-connected systems.

Hybrid systems have gained significant traction in tackling the challenge of electrification while also considering environmental considerations, as indicated in the existing body of literature. However, it's noteworthy that hybrid systems have yet to find implementation in certain developing nations, Cameroon being a case in point. This present study delves into the viability of a hybrid renewable energy system in Douala, which employs a combination of PV/battery/diesel and is integrated with the grid. The analysis was conducted using HOMER Pro, marking a pioneering endeavor in this domain. The integration of these elements in this configuration aims to bolster the system's reliability. Moreover, this research has taken into careful consideration three distinct consumer categories.

The subsequent sections of this document are organized as follows: Section “Materials and methods” delineates the materials and methodologies adopted for this study. Moving forward, Section “Results and discussion” unveils the results and ensuing discussions derived from the study's findings. Finally, Section “Conclusions and future research directions” encapsulates the study's outcomes and deliberates upon them in the concluding remarks.

## Materials and methods

Numerous commercial computational solutions are at your disposal for conducting a techno-economic assessment of renewable energy systems (RES) functioning both within on-grid and off-grid contexts. Among these options, when delving into a literature review that contrasts various computational tools, HOMER emerges as the most commonly employed software. This prevalence can be attributed to a variety of factors. Firstly, the software generates a graphical representation of efficiency, effectively presenting the results. Additionally, its user-friendly interface is designed for straightforward comprehension. Furthermore, the software streamlines the process of simulating and enhancing hybrid energy systems. Given its commercial availability, it is accessible to the general public, offering a free trial period as well. These aspects of accessibility hold particular significance for developing nations, where a majority of electricity-deprived communities are concentrated (references^26,27^). In the present study, HOMER was the software of choice for executing a techno-economic analysis.

HOMER Pro, an application developed by the NREL in the United States, serves the purpose of optimizing and conducting sensitivity analyses on proposed model projects. Its main objective is to identify the most viable hybrid model system for specific geographical locations. This determination hinges on two critical factors: the NPC and the LCOE. The software resource discussed in citation^28^ is recognized as the preeminent option for crafting hybrid renewable energy systems and executing comprehensive techno-economic assessments, regardless of whether these systems are integrated into the power grid or remain independent.

In its operational process, the software engages in a simulation of the system across a full annual cycle of 8760 h, subsequently evaluating the optimized results concerning the current Total Net Present Cost compared to the total net present cost. Homer's capabilities extend to identifying attributes of system components and approximating various costs, encompassing the overall lifetime expenditure, operational and maintenance costs, net current costs, and energy-related expenses. Additionally, the software accommodates the application of a Sensitivity Assessment technique, which facilitates the exploration of how variations in input parameters impact the resulting outputs^29^. The efficacy of Homer Pro software is governed by four primary constraints, known as simulation, optimization, sensitivity analysis, and appropriate component selection, as outlined in reference^30^. Prior to initiating the simulation process, a preliminary analysis is conducted to evaluate the requisite load, available resources, and the selection of various components. According to information presented in citation^31^, a comprehensive examination of data pertaining to rural areas and end-users is essential for the successful execution of energy initiatives. Following the conclusion of this assessment, the collected data is input into the software to yield outcomes that have undergone optimization and sensitivity analysis. These results are determined based on factors like net present cost and the lowest cost power rate, ensuring their viability.

**Figure 2:**
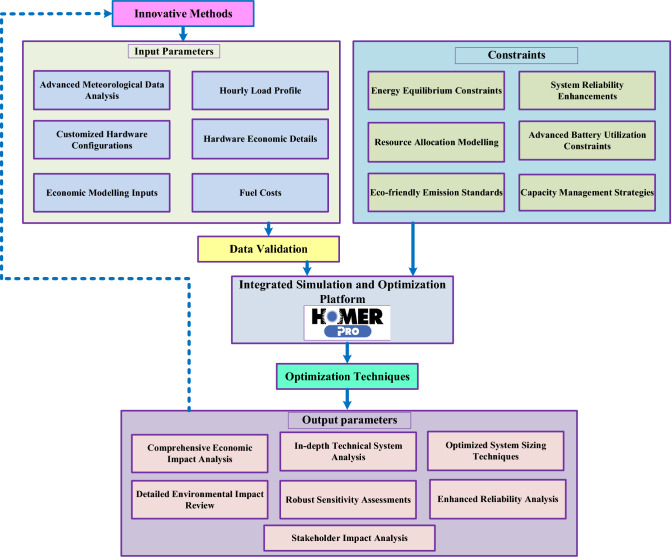
Research framework and innovations in hybrid renewable energy systems.

The dissemination of this information to stakeholders and investors aims to offer them a clearer comprehension of the potential benefits associated with the project. The procedural flowchart for this methodology is depicted in Fig. 2, visible below for reference.

### Metrological and social profile of the selected area

#### Study area

**Figure 3:**
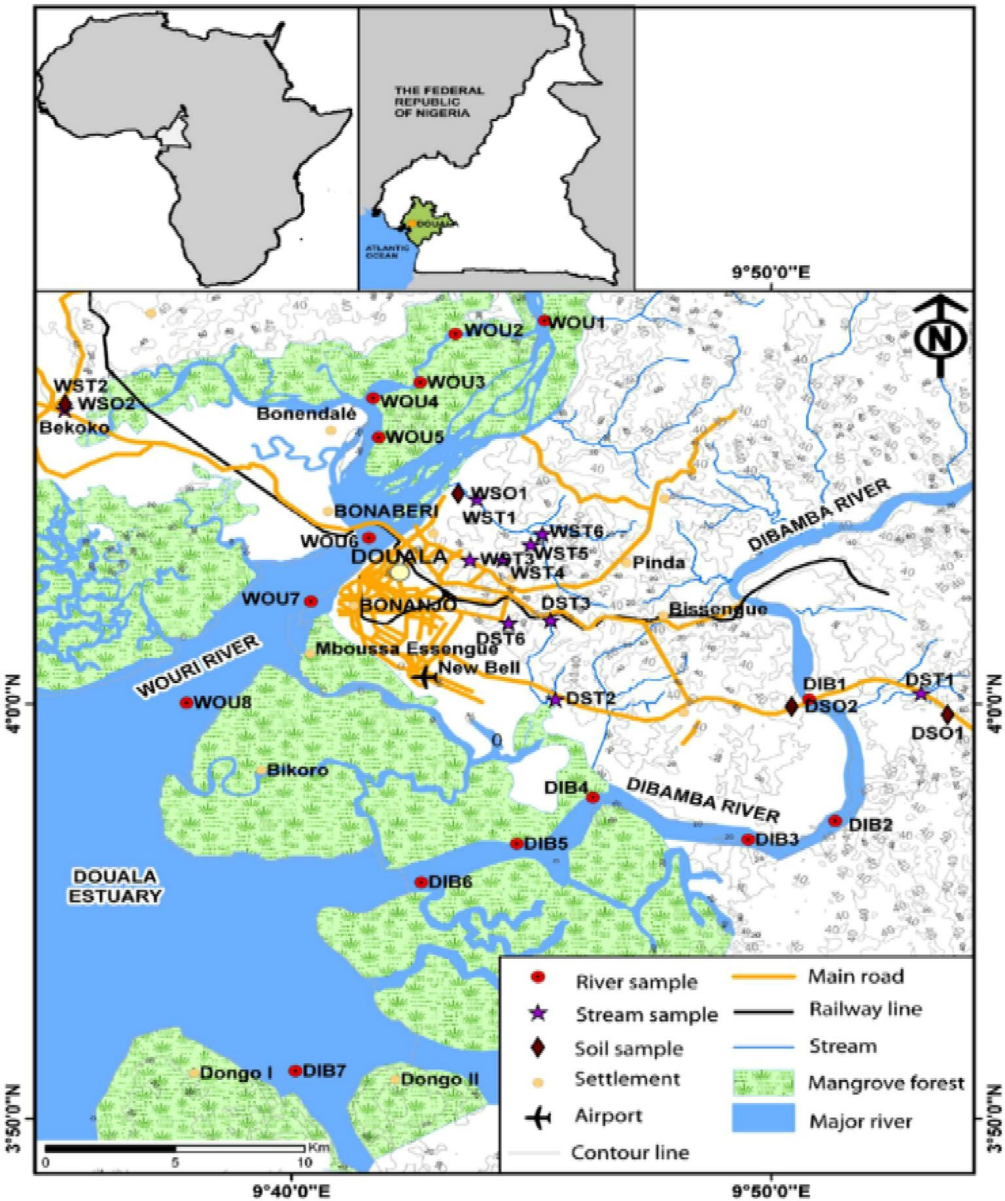
Site location of Douala, Cameroon.

The chosen location for this investigation is Douala, which serves as the economic capital of Cameroon, as shown in Fig. 3. Situated on the coast of the Wouri stream, the city's geographical coordinates are 4°3′53.77ʺ north of the equator and 9°41′15.41ʺ east of the Greenwich meridian. Its elevation reaches 13 m above sea level. Covering an urban area of around 210 square kilometers, Douala accommodates an estimated 3.6 million residents, resulting in a population density of approximately 177.79 individuals per square kilometer. The city undergoes four distinct seasons within the year. The initial season encompasses an extended period of rainfall from May to October. This is followed by a briefer dry season from November to December. Subsequently, a brief rainy season occurs in January and February. The final season, spanning from March to May, constitutes a lengthy dry period. As outlined in reference^19^, the average annual precipitation measures 963.7 mm, while the average temperature remains at 27.5 degrees Celsius. The choice of this location was based on a comprehensive assessment that revealed the lack of reliable and affordable electricity, despite the notable potential for renewable energy sources, particularly solar power. Ensuring a stable electricity supply is a crucial necessity for the country's progress, emphasizing the need for Douala to prioritize its accessibility.

#### Electrical load estimation

Examining the electrical load profile stands as a critical factor in conducting research. The focus of this study is a residential building positioned in Douala; a city situated in the coastal area of Cameroon. The process of gathering load data encompassed categorizing consumers into three separate groups, delineated by their daily consumption behaviors. Starting from June 1st, 2012, the electricity pricing structure, exclusive of taxes, set by Eneo Cameroon for low voltage customers is outlined in Table 1 as indicated by reference^32^.

**Table 1:** Monthly consumption bracket tariffs.

Domestic or residential uses		
Monthly consumption bracket tariffs		
Consumption less than or equal to 110 kWh (low)	Consumption between 111 and 400 kWh (medium)	Consumption between 401 and 800 kWh (high)
$0.1/kWh	$0.14/kWh	$0.17/kWh

The initial category consists of high consumers whose daily usage surpasses 5 kW. The subsequent category encompasses moderate consumers whose daily consumption falls between 3.3 and 5 kW. The final group comprises small consumers whose daily consumption remains below 3.3 kW. This classification was adopted by the electricity distribution company to assess the load pattern. In this study, random variability variables to increase the realism and take into consideration the uncertainties associated with home power consumption have been included. Among them were a 15% daily fluctuation and a 20%-time step variability. These changes were included to reflect the natural volatility and swings seen in home power usage on a daily and hourly basis.

**Figure 4:**
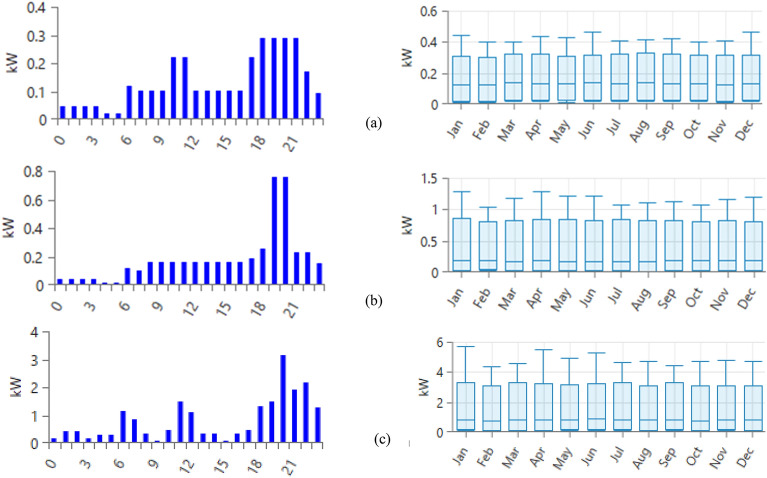
Different types of load profile depending on level of life (**a**) low consumer, (**b**) medium consumer, (**c**) large consumer.

Although our method aims to provide realistic representations, there is some degree of ambiguity due to the complexity of real-world use patterns. Tables 2, 3, and 4 provide estimations of consumers' daily usage based on specific requirements like lighting, ventilation, television, heating, and other pertinent factors. The load profile represented in Fig. 4 is formulated using the daily consumption data, which is computed and distributed across both a 24-h and an 8760-h timeframe.

**Table 2:** Estimated household high consumer.

Time	Household load											Energy consumed (W/h)
	Types of appliances/rating power (W)											
	Led lamp/10	TV/100	Electric iron/1000	Fan/60	Phone charger/5	Fridge/250	Lap top/45 W	Heater water/1000 W	Air conditioner/1700 W	Microwave oven/1000 W	Washing machines/500 W	
01:00	4				5	1	2					405
02:00	4				5	1	2					405
03:00	4				5		2					155
04:00	4					1						290
05:00	4					1						290
06:00	4	1								1		1140
07:00		1				1					1	850
08:00		1				1						350
09:00		1										100
10:00		1		2		1						470
11:00		1	1	2		1						1470
12:00		1	1									1100
13:00		1				1						350
14:00		1				1						350
15:00		1										100
16:00		1				1						350
17:00		1		2		1						470
18:00	12	1		2				1				1340
19:00	12	1				1		1				1470
20:00	12	1				1			1	1		3170
21:00	12	1							1			1920
22:00	12	1				1			1			2170
23:00	12	1			5	1	2		1			2285
00:00	4				5		2					155
Total (Wh/day)												21,155

**Table 3:** Estimated household medium consumer.

Time	Household load					Energy consumed(W/h)
	Types of appliances/rating power (W)					
	Led lamp/10	TV/100	Electric iron/500	Fan/60	Phone charger/5	
01:00	2				5	45
02:00	2				5	45
03:00	2				5	45
04:00	2					20
05:00	2					20
06:00	2	1				120
07:00		1				100
08:00		1		1		160
09:00		1		1		160
10:00		1		1		160
11:00		1		1		160
12:00		1		1		160
13:00		1		1		160
14:00		1		1		160
15:00		1		1		160
16:00		1		1		160
17:00		1		1	5	185
18:00	7	1		1	5	255
19:00	7	1	1	1	5	755
20:00	7	1	1	1	5	755
21:00	7	1		1		230
22:00	7	1		1		230
23:00	7			1	5	155
00:00	2				5	45
Total (Wh/day)						4625

**Table 4:** Estimated household low consumer.

Time	Household load				Energy consumed(W/h)
	Types of appliances/rating power (W)				
	Led lamp/10	TV/100	Fan/60	Phone charger/5	
01:00	2			5	45
02:00	2			5	45
03:00	2			5	45
04:00	2				20
05:00	2				20
06:00	2	1			120
07:00		1			100
08:00		1			100
09:00		1			100
10:00		1	2		220
11:00		1	2		220
12:00		1			100
13:00		1			100
14:00		1			100
15:00		1			100
16:00		1			100
17:00		1	2		220
18:00	7	1	2		290
19:00	7	1	2		290
20:00	7	1	2		290
21:00	7	1	2		290
22:00	7	1			170
23:00	7			5	95
00:00	2			5	45
Total (Wh/day)					3225

#### Assessment of renewable resources

**Figure 5:**
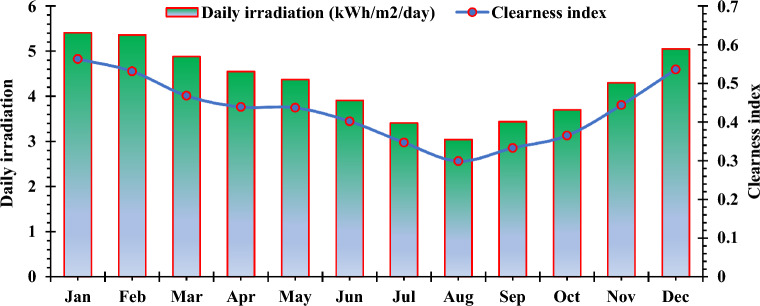
Solar daily radiation and clearness index at the selected location.

The evaluation of the potential energy resources primarily relies on the geographical coordinates of the designated area. Within the country of Cameroon, a noteworthy capacity for solar energy exists. The resource potential of the proposed location has been assessed based on its geographic positioning. The analysis drew upon meteorological data from NASA's Surface Meteorology and Solar Energy Database, which was utilized in HOMER. Mean monthly profiles of solar irradiation and clearness index are depicted in Fig. 5. Douala experiences an average annual solar radiation of 4.29 kWh/m^2^/day, reaching its peak in January and its nadir in August, registering 5.41 and 3.04 kWh/m^2^/day, respectively. The solar brightness index spans from 0.29 in August (characterized as extremely overcast) to 0.56 in January (signifying very sunny conditions), indicating a robust potential for solar energy in the examined region.

#### Temperature

**Figure 6:**
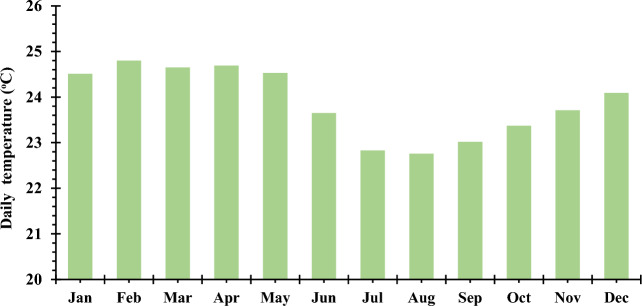
The monthly mean temperature at the location.

The efficiency of a photovoltaic system is notably influenced by temperature, leading to a decrease in power production. The temperature data sourced from a solar power installation situated at the Douala University Institute of Technology throughout the year 2020 is presented in Fig. 6. The average annual temperature for this specific site stands at 26.83 °C. The highest documented temperature, reaching 28.79 °C, was recorded in the month of February.

### Hybrid system components

**Figure 7:**
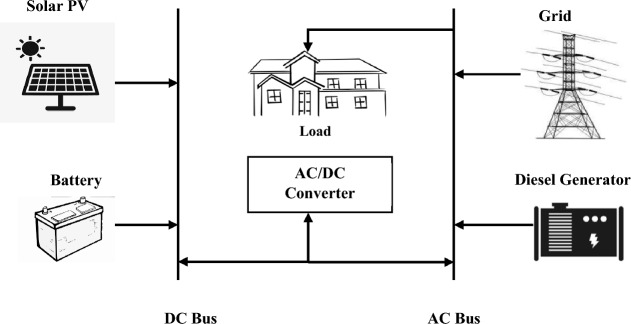
HOMER grid-connected hybrid system schematic.

Incorporating a range of energy sources into the grid-connected electricity generation process is recognized as a dependable strategy for enhancing the technical and economic aspects of hybrid systems. The primary challenge when configuring such systems lies in effectively sizing energy components, a task influenced by both energy costs and overall system efficiency. The hybrid power generation system employed in this study is illustrated in Fig. 7. The modeling process for this project was executed using HOMER Pro software. This modeling approach involved the integration of diverse elements, including solar photovoltaic (PV) systems, diesel generators, batteries, grid connections, and converters. This section presents a mathematical model encompassing the distinct components of the proposed Hybrid Renewable Energy System (HRES), along with technical and economic specifications. Prior to engaging in system sizing and optimization, undertaking this particular step is crucial as it offers valuable insights into the performance of individual system elements across various operational scenarios.

#### Solar PV modeling

The solar panel stands as a crucial component within a solar energy system^33^. Within the solar panel, the photovoltaic cells undertake the conversion of direct incoming radiation into direct current. As outlined in reference^34^, solar energy represents a form of renewable energy with the capability to address the global energy demand. Given Cameroon's considerable solar potential, it is regarded as the most fitting renewable energy solution to prioritize, as stated in reference^35^. The mathematical assessment of the photovoltaic (PV) system's power output is conducted via the HOMER software. This evaluation is grounded in the influence of solar radiation and temperature, both of which are factored in using the Eq. (1) provided in reference^29,36^.

1 $${\text{P}}_{\text{PV}}={\text{y}}_{\text{PV}}\times {\text{f}}_{\text{PV}}\times \left(\frac{{\overline{\text{G}} }_{\text{T}}}{{\overline{\text{G}} }_{\text{T},\text{STC}}}\right)\times \left[1+{\alpha }_{\text{P}}\right.\left.\left({\text{T}}_{\text{C}}-{\text{T}}_{\text{C},\text{STC}}\right)\right]$$

where ${T}_{C}$ is the cell temperature which is determined by the Eq. (2).

2 $${\text{T}}_{\text{C}}={\text{T}}_{\text{amb}}+\left(0.0256\times \text{G}\right)$$

where, ${y}_{PV}$ is the rated power of the PV array under standard test conditions [kW], ${f}_{PV}$ is the PV derating factor [%], ${\overline{G} }_{T}$ is the solar radiation incident on the PV array in the current time step [kW/m^2^], ${\overline{G} }_{T, STC}$ is the incident radiation at standard test conditions [1 kW/m^2^], ${\alpha }_{P}$ is the temperature coefficient of power [%/^0^C], ${T}_{C}$ is the PV cell temperature in the current time step [°C], ${T}_{C, STC}$ is the PV cell temperature under standard test conditions [°C]. The solar PV module with the technical and economic inputs used in this study is presented in Tables 5 and 9.

**Table 5:** Technical parameters specifications for the PV module^37^.

Component	Detail	Value
Solar PV	Rated power (kW)	1
	Normal operating cell temperature (°C)	47
	Efficiency at STC (%)	15
	De-rating factor (%)	80
	Ground reflectance (%)	20
	Lifetime (years)	25

#### Diesel generator (DG)

The diesel generator module functions as a supplementary energy source for the HRES when the load demand isn't met by the RES or energy storage system. The assessment of this module's efficacy commonly hinges on factors such as its efficiency, the type of fuel employed, and the amount of fuel consumed. The hourly fuel consumption of a diesel generator set is determined through a linear model, which is expounded upon in Eq. (3) as detailed in reference^35^.

3 $${F}_{dg}={B}_{g}\times {P}_{dg}+{A}_{g}\times {P}_{dg-out}$$

where ${P}_{dg-out}$ is the DG output power, ${P}_{dg}$ is the DG rated power, ${A}_{g}$ and ${B}_{g}$ are constant representing the coefficient of fuel consumption which approximately the values 0.246179 L/kW and 0.08415 L/kW respectively. The technical specifications for the DG are given in Table 6.

**Table 6:** Technical parameters specifications for the diesel generator^37^.

Component	Detail	Value
Diesel generator	Lower heating value (MJ/kg)	43.2
	Density (kg/m^3^)	820
	Carbon content (%)	88
	Sulfur content (%)	0.4
	Carbon dioxide (g/L fuel)	16.5
	Nitrogen oxides (g/L fuel)	15.5
	Unburned hydrocarbons (g/L fuel)	0.72
	Particulate matter (g/L fuel)	0.1
	Sulfur dioxide (%)	2.2

#### Battery storage

In the realm of system modeling, batteries function as reservoirs for surplus energy generated by renewable sources. When the energy generation surpasses the system's immediate demand, these batteries accumulate the excess energy. Conversely, in instances where the system's energy requirement outpaces production, the batteries channel the stored energy back into the system. This stored and transferred energy maintains an alternating current (AC) voltage configuration.

The influence of the quantity of batteries on the system is of paramount importance, owing to the substantial expenses associated with these battery units^38^. The cost for each unit of battery, capable of storing 1 kWh within the system, is projected at $450. This cost aligns with the cost of renewal. The simulation also accounts for a comprehensive operational and maintenance cost totaling $20. The projected lifespan of the battery falls within the range of 15 to 25 years. Concurrently, the simulation highlights a battery depth of 80%.

Equations (4) and (5) as presented below^24,39^, encapsulate the state of battery charging in the simulation.

4 $${\text{E}}_{\text{b}}\left(\text{t}+1\right)={\text{E}}_{\text{b}}\left(\text{t}\right)\times \left(1-\upsigma \right)-\left(\frac{{\text{E}}_{\text{l}}\left(\text{t}\right)}{{\upeta }_{\text{cnv}}}-{\text{E}}_{\text{g}}\left(\text{t}\right)\right)\times {\upeta }_{\text{BD}}$$

5 $${E}_{b}\left(t+1\right)={E}_{b}\left(t\right)\times \left(1-\sigma \right)-\left({E}_{g}\left(t\right)-\frac{{E}_{l}\left(t\right)}{{\eta }_{cnv}}\right)\times {\eta }_{BC}\boldsymbol{ }\boldsymbol{ }$$

where ${E}_{l}\left(t\right)$ and ${E}_{g}\left(t\right)$ are the energy demand and generated power, respectively. ${\eta }_{BD}$ and ${\eta }_{BC}$ represent the discharge and charge efficiencies of the battery. The parameter $\sigma$ is the self-discharge of the battery. ${\eta }_{cnv}$ is the efficiency of the converter. Technical parameters and specifications for the battery are shown in Table 7**.**

**Table 7:** Technical parameters and specifications for the battery.

Component	Detail	Value
Battery	Nominal voltage (V)	12
	Nominal capacity (kWh)	1.7
	Maximum capacity (Ah)	141
	SOC limits (%)	20–100
	Roundtrip efficiency (%)	80
	Capacity ratio	0.363
	Maximum charge current (A)	21.8
	Maximum discharge current (A)	230
	Lifetime throughput (years)	5

#### Grid

The State Electricity Board, overseen by the Department of Power within the Government of Cameroon, is responsible for delivering electricity to the public at an approximate cost of $0.1 per kilowatt-hour (kWh). However, this supply is plagued by frequent interruptions, rendering it notably unreliable. The distribution station is located in close proximity to residential zones, yet the absence of precise information concerning power grid disturbances is a prevailing issue.

#### Bi-directional inverter system

In order to ensure an uninterrupted transfer of energy between AC and DC buses, the integration of a power combiner becomes necessary^40,41^. Within HOMER, the converter component serves a dual purpose: it operates as an inverter, facilitating the conversion from DC to AC, and as a rectifier, enabling the conversion from AC to DC. The recently incorporated converter in the system was subjected to simulation as a standard converter defined within the HOMER platform. The efficiency of this converter can be ascertained using Eq. (6), as detailed in reference^42^.

6 $${\eta }_{cnv}=\frac{{P}_{output}}{{P}_{input}}$$

where ${P}_{output}$ stands for the output power from/to the converter, where ${P}_{input}$ is the input power from/to the converter. The technical and economic parameters and specifications of the converter used in the system are listed in Tables 8 and 9.

**Table 8:** Technical parameters specifications for the converter.

Component	Detail	Value
Bi-directional converter	Relative capacity (%)	90
	Lifetime (years)	15
	Efficiency (%)	95

**Table 9:** Cost specifications of the system components.

Component	Capital cost	Replacement cost	O&M cost
PV	950$/kW	800$/kW	10$/kW
Diesel generator	250$/turbine	160$/turbine	0.05$/turbine/year
battery	250$/battery	200$/battery	2$/battery/year
Bi-directional converter	712$/kW	550$/kW	10$/kw/year

### Problem formulation

The HOMER software conducts simulations encompassing all potential system configurations within the predefined exploration range, resulting in an optimized grid system. The initial phase of this procedure involves constructing an optimization challenge, which encompasses the identification of an objective function.

#### Objective function

The aim of this research is to calculate the Net Present Cost (NPC) and Cost of Energy (COE) related to Eqs. (7) and (10) correspondingly while taking into account the constraint of reliability in Integrated Renewable Energy.

##### Net present cost (NPC)

The NPC of a component is calculated as the difference between its present value of costs over the life of a project and its present value of revenues during the same period. The evaluation process can be performed using Eq. (7) as described in references^37,38^.

7 $${C}_{NPC}=\frac{{C}_{ann,tot}}{CRF\left({i, R}_{proj}\right)}$$

The total annualized cost includes annual operation and maintenance (O&M) cost, emissions cost, and annual fuel (generator) cost. $CRF\left({i, R}_{proj}\right)$ is a function that delivers the return on capital (CRF). The annual effective discount rate [%], and the duration of the project ${R}_{proj}$ [year] is determined by the following equation^43^.

The Capital Recovery Factor CRF is determined by Eq. (8) ^44–46^.

8 $$CRF\left({i, R}_{proj}\right) =\frac{i{\left(1+i\right)}^{\tau }}{{\left(1+i\right)}^{\tau }-1}$$

where τ stands for the life span of the plant, considered as 25 years in the study.

##### Net discount rate ($i$)

The net discount rate in HOMER Pro is defined as the actual interest rate utilized for the conversion of costs between annualized and in-time values. The net interest rate is calculated by HOMER using the following Eq. (9) ^44^.

9 $${\varvec{i}}=\frac{{{\varvec{i}}}^{\boldsymbol{^{\prime}}}-{\varvec{f}}}{1+{\varvec{f}}}$$

The formula mentioned above represents the nominal interest rate in conjunction with the annual inflation rate. According to source^47^, the inflation and net discount rates for January 2023 in Cameroon are 3.25% and 4.20% respectively.

##### Cost of energy (COE)

The Levelized Cost of Electricity (LCOE), commonly referred to as COE, is a crucial metric in the energy industry^48^. It represents the average cost per kilowatt-hour (kWh) of electrical energy generated by a facility. To calculate the COE, the annual cost of electricity generation is divided by the total power load. This calculation is performed using the Eq. (10) ^42^.

10 $$COE={C}_{NPC}\times CRF/\sum_{t=1}^{8760}{E}_{gen}({\$}/\text{kWh})$$

where ${E}_{gen}$ stands for the total energy generated by both the grid and the microgrid (MG). It is obtained by Eq. (11)**.**

11 $${E}_{gen}={E}_{ge{n}_{MG}}+{E}_{ge{n}_{grid}}$$

where ${E}_{ge{n}_{MG}}$ stands for the energy generated by the microgrid whereas ${E}_{ge{n}_{grid}}$ is the energy generated by the grid only.

#### Unmet load

The term "uncovered load" refers to the annual uncovered load-to-total annual load ratio, which can be calculated using Eq. (12) provided in references^45,49^.

12 $$Unmet Load=\frac{Annual \, Non-served \, Load}{Annual \, Entire \, Load}$$

### Model constraints

#### Reliability of the system

System reliability pertains to a system's ability to uphold its operational capabilities amid brief power disruptions, typically spanning a few hours^50^. Throughout the year, there might be several occurrences of these service interruptions. The ability to sustain functionality during extended outages is regarded as a facet of resilience. In scenarios where power is cut off, systems equipped with energy storage or backup generators can continue functioning^51^. The concept of the probability of power supply loss (LPSP) is employed to evaluate the robustness of the system.

#### Renewable energy penetration

The Renewable Energy Penetration (REP) serves as a measure depicting the percentage of energy generated from renewable sources within a specific system. HOMER software employs Eq. (13), expounded upon in reference^52^, to compute this metric.

13 $$REP\left(\%\right)=\left(1-\frac{\sum {P}_{G}}{\sum {P}_{ren}}\right)\times 100$$

where P_G_ [kWh/year] is the electricity production from Grid, ${P}_{ren}$ is the overall renewable electrical energy (kWh/year).

#### Battery storage constraint

The battery operation is subject to two conditions, namely the state of charge (SOC) boundaries for overcharging and over-discharging. The SOC must be maintained within the safe limits as specified in Eq. (14) ^53^. At every time step t, the battery bank's capacity ${E}_{b}\left(t\right)$ falls within a range of minimum and maximum capacity. The Depth of Discharge (DOD) of a battery is determined by its technology. The DOD refers to the percentage of the battery's capacity that has been discharged during use. The constraint is represented by Eq. (15) as cited in reference^54^.

14 $${SOC}_{min}\le SOC\left(t\right)\le {SOC}_{max}$$

15 $${E}_{bmin}\le {E}_{b}\left(t\right)\le {E}_{bmax}$$

16 $${E}_{bmin}=\left(1-DOD\right){\times E}_{bmax}$$

### Power’s constraint

Equation (17) represents the constraints for power balance and battery charge or discharge.

17 $$\sum_{j=1}^{N}{P}_{PV}+{P}_{Gen}+{P}_{grid}\pm {P}_{BBS}-{P}_{load}=0$$

where ${P}_{PV}$ corresponds to the PV power output ${P}_{gen}$ is the diesel generator power, ${P}_{grid}$ is the power utility, and ${P}_{BBS}$ is the battery system power. If ${P}_{BBS}$ is positive, the battery is discharging and ${P}_{BBS}$ is negative during the charging mode^55,56^.

## Results and discussion

### Optimization results

The current research employed the HOMER software to simulate and analyze a hybrid energy system comprising a solar panel, diesel generator, battery, and grid connection. The simulation provided data for unit energy cost and net present cost. The optimization outcomes are assessed preceding the techno-economic analysis findings. Moreover, the consumer classification into three types takes into consideration the utility tariff. Various parameters are considered for the technical evaluation of the system. The anticipated lifespan for the proposed microgrid (MG) system is assumed to be 25 years. Additionally, all scenarios will feature uniform components within the MG system. Table 10 displays the optimal cost outcomes for these scenarios.

**Table 10:** Optimization results.

Detail	Component	Unit	Most optimized result		
			Large-consumer	Medium-consumer	Low-consumer
System configuration	Solar panel	kW	11.8	1.5	0.956
	Battery	quantity	8	1	1
	Diesel generator	kW	5.80	1.4	0.530
	Bi-directional converter	kW	3.98	0.635	0.406
	Dispatch strategy	LF or CC	LF	LF	LF
Power output	Solar PV	kW/year	11,703	1492	951
	Diesel generator	kW/year	36.7	32.6	2.92
	Grid purchases	kW/year	5111	976	694
	total	kW/year	16,851	2500	1648
	Unmet load	kW/year	0	0	0
Cost	COE	US$/kWh	0.06206	0.08793	0.07930
	NPC	US$/kW	23,709.21	5451.69	3271.60

**Figure 8:**
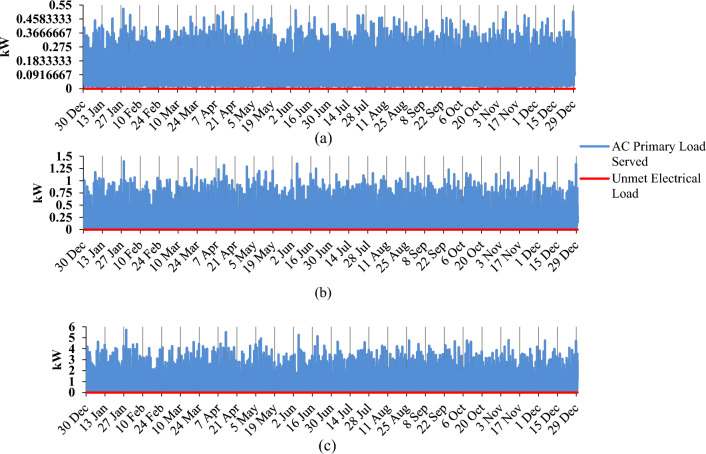
Monthly unmet load: (**a**) low consumer; (**b**) medium consumer; (**c**) large consumer.

Assessing the reliability of the Hybrid Renewable Energy System (HRES) necessitates employing a pivotal parameter tailored to the particular context. The system possesses the potential to produce top-notch energy and fulfill the prescribed load requirements. The study showcases findings that exemplify the optimal model's capacity to fulfill all demands. Figure 8 visually depicts the absence of unmet load across the three dimensions of the issue. Subsequent to this juncture, enhancements have been introduced to the system to bolster its reliability and efficiency.

### Production distribution of system components

**Figure 9:**
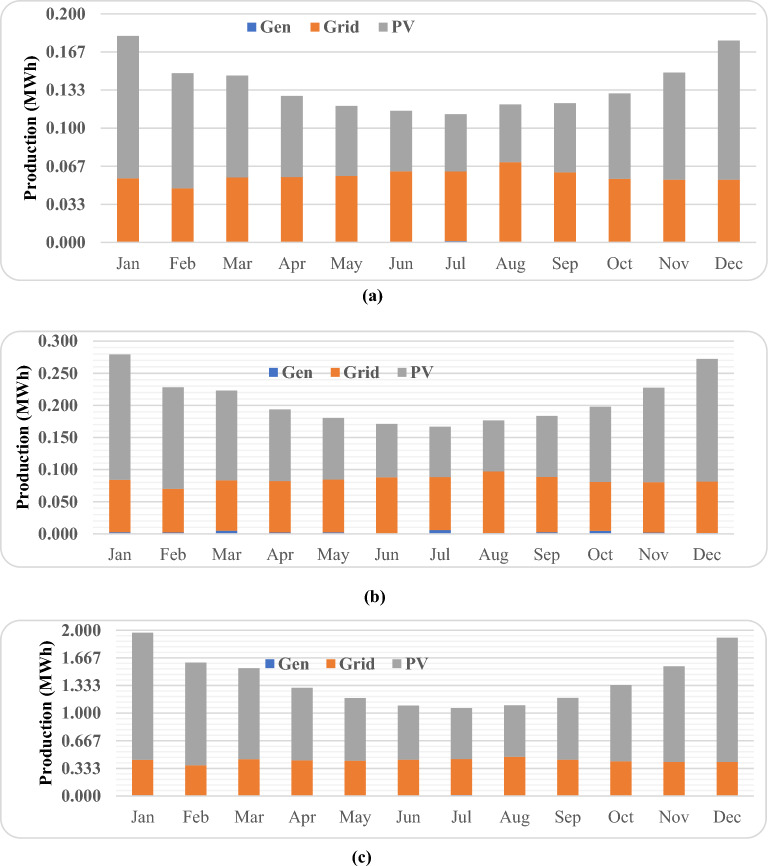
Monthly average electricity production for optimal HRES: (**a**) low consumer; (**b**) medium consumer; (**c**) large consumer.

The evaluation process takes into account the distribution of system components within the overall energy production framework. Figure 9 provides a visual representation of the monthly energy output from the diverse elements of the system. In the three scenarios, solar panels and the grid play a substantial role in power generation. It's evident from Fig. 9 that solar panels and the grid contribute significantly to the energy generation process. The input from diesel generators, on the other hand, holds a relatively marginal influence on energy production. The surplus annual electricity generation for low, medium, and high consumers is documented at 88.5 kWh, 158 kWh, and 2204 kWh, respectively. As highlighted in Fig. 8, the annual deficit in electrical load is 0 kWh. It's noteworthy that in this specific configuration, not all sources operate concurrently. Depending on the load requirements, a combination of multiple sources may be utilized to bridge any supply shortfall.

### Solar panels impact the system

**Figure 10:**
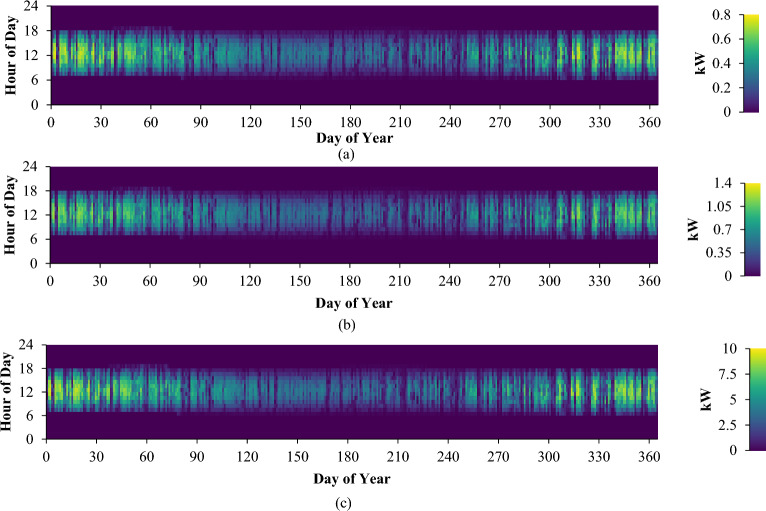
Solar panel monthly average electricity production: (**a**) low consumer; (**b**) medium consumer; (**c**) large consumer.

Figure 10 illustrates the power output values acquired from the solar panels integrated into our system under varying loads across different days and times throughout the year. Owing to the geographical location of the chosen region, the solar panels stand as the predominant energy source for the system. Additionally, the heightened capacity of these panels facilitates the rapid generation of a substantial energy volume within a short duration.

Simulation outcomes underscore uniformity across all three consumer categories in terms of the total annual operation, capacity factor, and unit cost of the electricity generated by the solar panels. Specifically, the annual operation spans 4389 h, the capacity factor stands at 11.3%, and the energy unit cost is $0.0439/kWh. However, it's imperative to consider certain nuanced particulars.

For consumers with low power usage, the annual energy production rests at 951 kWh. Daily, the solar panels generate an average of 2.60 kWh, attaining a PV penetration rate of 80.6%. Notably, these solar panels within the system exhibit a maximum capacity of 0.956 kW.

Moving to medium consumers, the solar panels contribute to a yearly electrical energy output of 1492 kWh, averaging a daily production of 4.09 kWh. The photovoltaic (PV) penetration rate for this category stands at 91.8%, while the solar panels' maximum capacity reaches 1.50 kW.

High consumers, on the other hand, have the capability to generate an annual total of 11,703 kWh of electrical energy. With an average daily output of 32.1 kilowatt-hours (kWh), the photovoltaic (PV) penetration rate reaches 98%. The solar panels integrated into the system boast a maximum capacity of 11.8 kW.

As indicated by Fig. 10, there are instances during specific times of the day when solar panels produce inadequate electricity. The primary energy source driving the system originates from the solar panels, predominantly due to the geographic attributes of the selected area.

### Effect of the grid on the system

**Figure 11:**
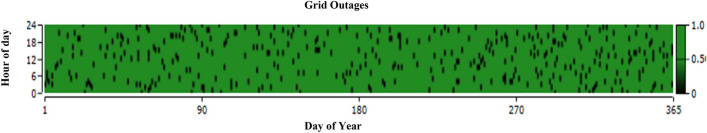
Yearly profile grid outages.

The envisaged HRES adopts a simulation-centric methodology for evaluating reliability. This simulation hinges on the occurrence of power outages, with an average annual frequency of 365 instances. Each outage necessitates approximately 2 h for repairs, as depicted in Fig. 11. Throughout the duration of these outage intervals, there is a cessation of electrical energy exchange between the grid and all relevant stakeholders.

**Figure 12:**
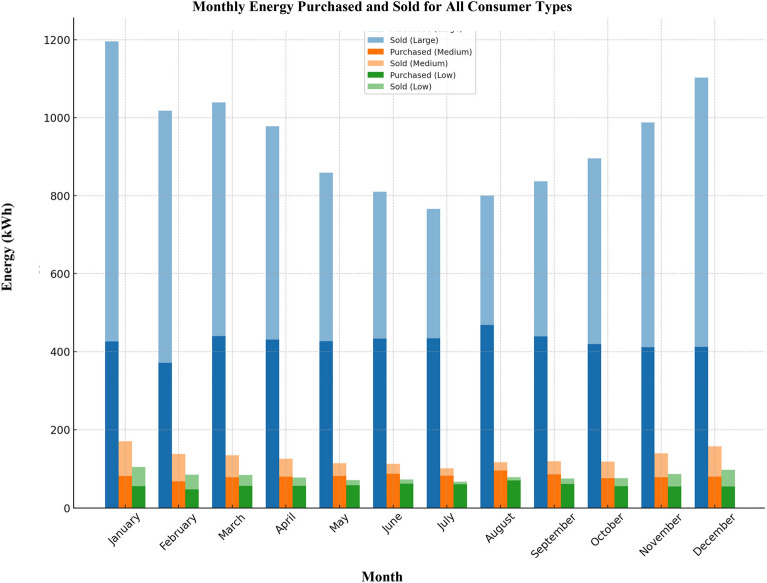
Annual energy purchased and sold by the grid.

The yearly energy acquired from and fed into the grid for the assorted scenarios under analysis are depicted in Fig. 12. In situations of substantial energy utilization, the grid provides a total of 5111 kWh, while a corresponding 6179 kWh is contributed back to the grid annually. For a moderate consumer, the annual energy intake from the grid stands at 976 kWh, with 573 kWh being supplied back to the grid. In the case of a low-energy consumer, the yearly grid procurement amounts to 694 kWh, accompanied by an annual grid injection of 283 kWh.

### Impact of the diesel generator on the system

**Figure 13:**
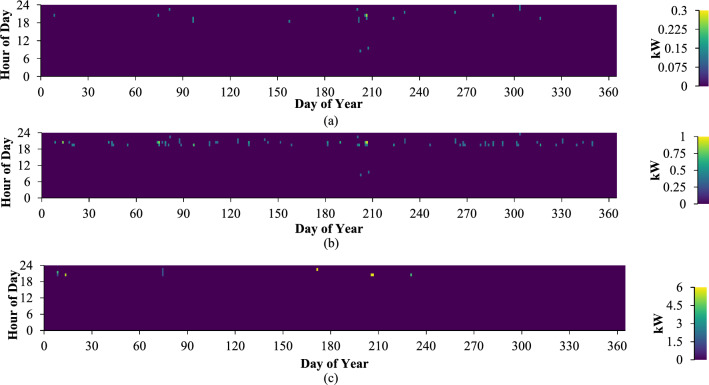
Diesel generator output power: (**a**) low consumer; (**b**) medium consumer; (**c**) large consumer.

Incorporating diesel generators into the system has led to improved reliability of the system and reduced project costs. The different groups of consumers are linked with three distinct load categories based on the prevailing electricity rates. As shown in Fig. 13, the diesel generator showed its highest energy output during nighttime hours. Additionally, a significant portion of the power generation was supplied to the end-user during rainy periods.

The diesel generator integrated into the system was employed by low-consumption users a total of 17 times annually, with a cumulative operational time of 21 h each year. In this usage, the generator generated a total of 2.92 kWh of electrical energy. The specific fuel consumption of the generator stands at 0.449 L per kilowatt-hour (L/kWh). Similarly, the diesel generator incorporated in the system was utilized 58 times per year by medium-consumption users, operating for a total duration of 77 h annually. The collective electrical energy produced by this generator amounted to 32.6 kWh. Its specific fuel consumption is measured at 0.421 L per kilowatt-hour (kWh).

The diesel generator incorporated in the system was utilized by heavy consumers for a total of 7 instances annually, with a runtime of 10 h per year, resulting in the generation of 36.7 kWh of electrical energy. The generator's specific fuel consumption is 0.324 L per kilowatt-hour (L/kWh). The diesel generator system's monthly power production is shown in Fig. 13.

### Effects of using batteries in the system

Integrating batteries into the system led to a significant increase in the total system cost. Table 11 illustrates the net present values and unit energy prices for three different consumer categories, along with the respective quantity of batteries they have incorporated.

**Table 11:** NPC and COE of the systems.

Consumer	Numbers of batteries	NPC ($)	COE($/kWh)
High	8	23,709.21	0.06206
Medium	1	5451.69	0.08793
Low	1	3271.60	0.07930

**Figure 14:**
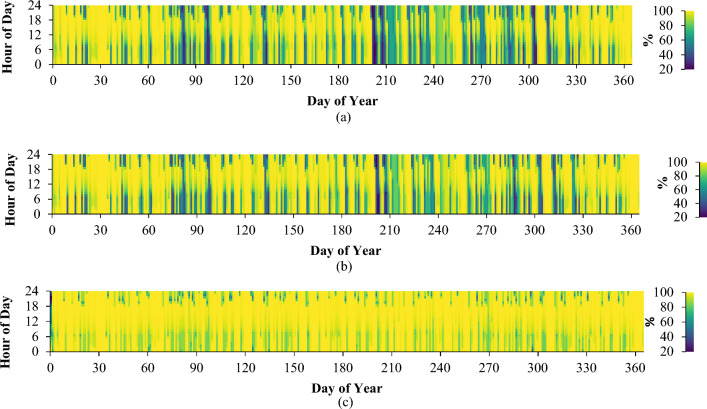
Battery charge status during the year: (**a**) low consumer; (**b**) medium consumer; (**c**) large consumer.

The rise in the number of batteries is clearly associated with a notable escalation in the system's expenses. In cases where there are 8 batteries, consumers with high usage display a net present cost (NPC) amounting to $23,709.21. In contrast, consumers with low usage and just 1 battery show an NPC of $3271.60. According to the presented data, the net present value demonstrates an inverse relationship with the quantity of batteries. The hourly state of charge of the integrated batteries throughout the year is depicted in Fig. 14.

### Sensitivity analysis

**Figure 15:**
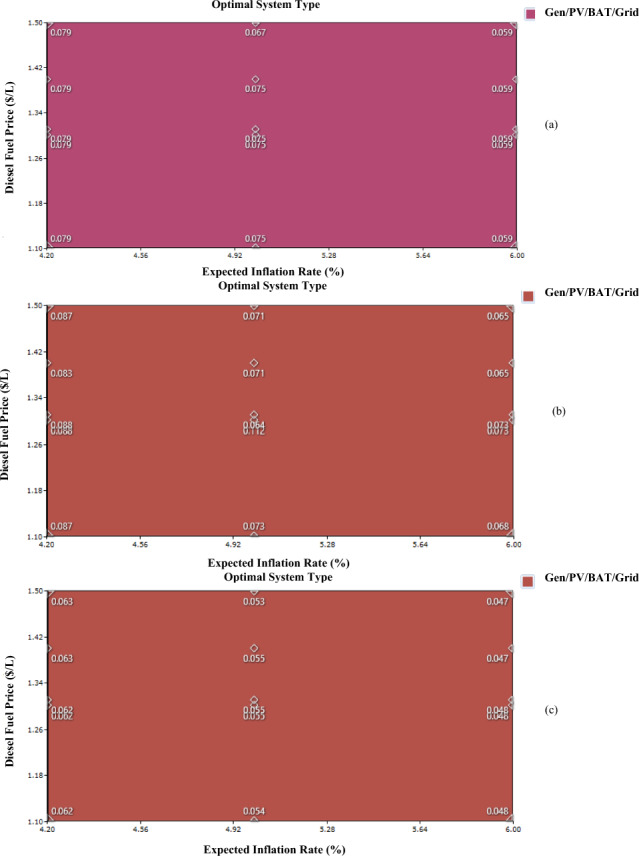
Sensitivity analysis: (**a**) Low consumer; (**b**) Medium consumer; (**c**) Large consumer.

The effect of changes in fuel costs and inflation is shown in the Fig. 15. In fact, by being aware of these two factors, investors and policymakers may better inform their choices when it comes to investing in the renewable energy sector. Area graphs representing energy prices were analyzed in this research for consumer groups classified as low (a), medium (b), and high (c). The surface in the aforementioned numbers shows the energy cost as it varies during the project term due to changes in fuel prices and inflation rates. In summary, all consumer categories see a drop in energy costs when the rate of inflation rises. It has also been noted that rising gasoline prices translate into rising energy expenses. The Cameroonian government might cut fuel costs and provide a 0% interest rate on cash spent in enabling the public to access power in order to encourage investment in renewable energy. The authors can also see that whatever the variation in the rate of inflation and the price of fuel, the optimum system remains the system including solar photovoltaic (PV) systems, diesel generators, batteries, grid connections.

### Cost-benefits analysis

**Figure 16:**
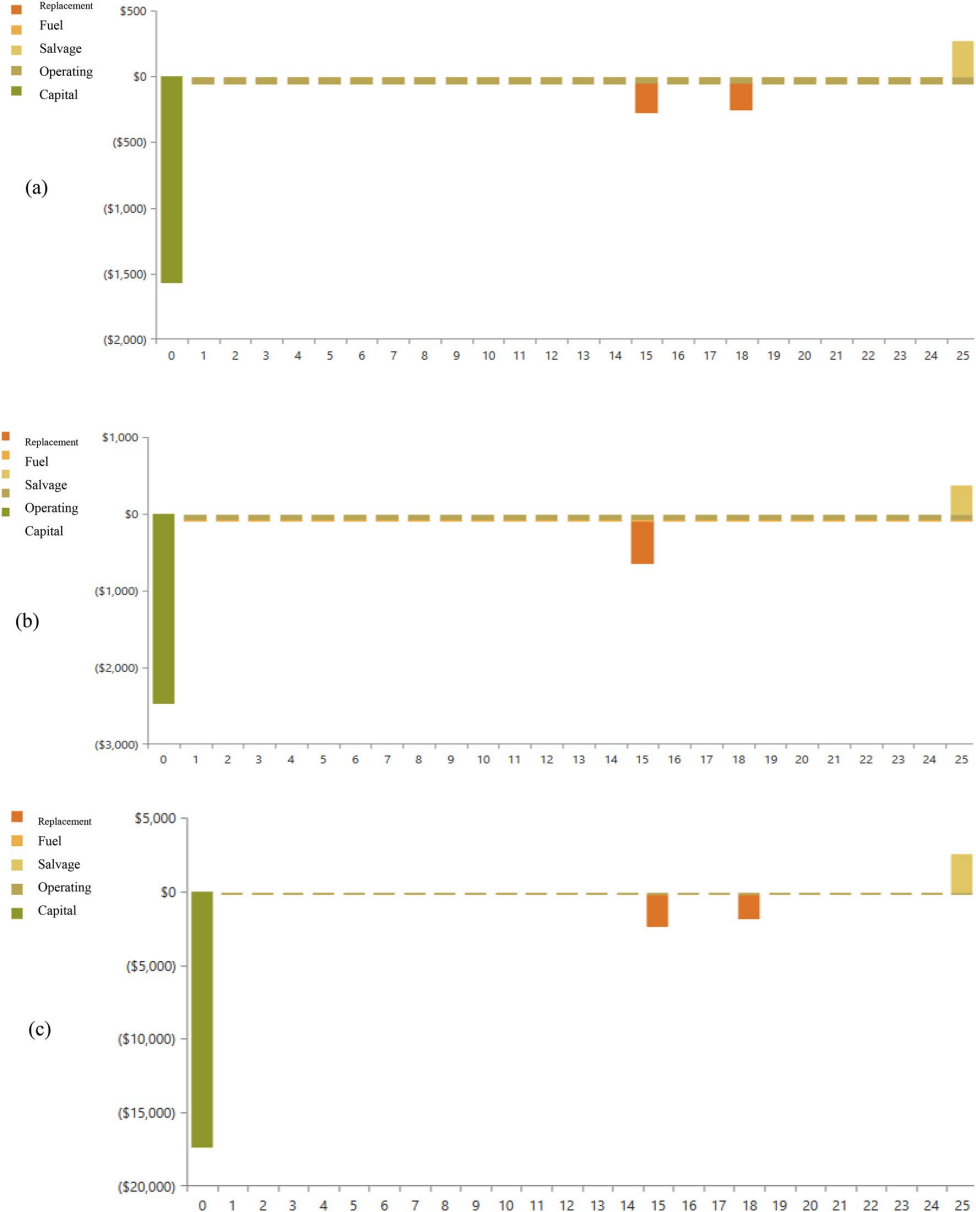
Nominal cash flow results: (**a**) low consumer; (**b**) medium consumer; (**c**) large consumer.

The cost analysis of the simulation results for the various consumers is presented in the form of bar graphs and detailed explanations in the Fig. 16. The investment costs, replacement costs and operation and maintenance (O&M) costs for each simulation are presented on the basis of the schematic design of the renewable energy system in the figure. According to the graphs, a value pointing upwards means a cash outflow, while a value pointing downwards means a cash inflow.

**Figure 17:**
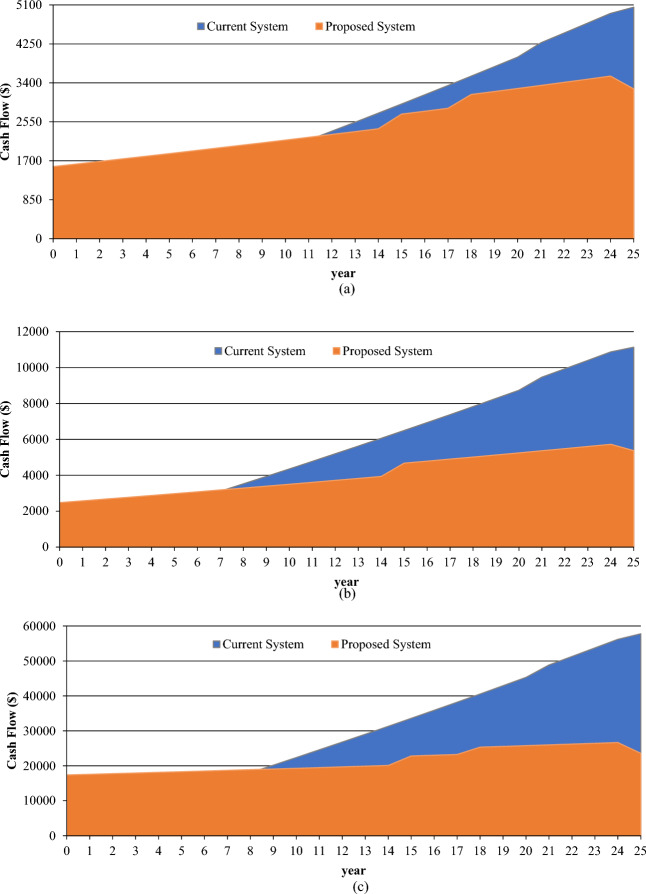
Cumulative cash flow over project lifetime: (**a**) low consumer; (**b**) medium consumer; (**c**) large consumer.

For each of the consumer groups in the basic scenario and the lowest-cost system scenario, Table 12 and Fig. 17 offers a thorough summary of the cost summaries and economic metrics.

**Table 12:** Comparative summary for different tariffs.

Categories	Cost summary		Economic metrics		
	Base case ($)	Lowest cost system ($)	IRR (%)	ROI (%)	Simple payback (year)
Low-consumer	27,096	3266	62	58	1.6
Medium-consumer	11,134	5370	12	9.1	7.5
Large-consumer	57,784	23,579	10	7.1	8.8

First off, the lowest-cost solution for every consumer group is much less expensive than the baseline scenario, which just includes the public grid and the diesel generator. Specifically, the "low consumption" category shows a notable decrease from $27,096 to $3266; this indicates a strong level of cost optimization.

The internal rate of return (IRR) values for each category-low, medium, and high consumption-are all positive when considering the economic characteristics. This implies that every consumer segment will get a positive return on investment (ROI). The ROI, which range from 7.1 to 58%, are also competitive and show promising economic outcomes.

Regarding the basic payback term, the durations for all categories range from 1.6 to 8.8 years, which are reasonably short. This suggests higher initial investment returns, consistent with the focus on shorter payback periods as beneficial economic factors.

### Environmental and emissions impact analysis

Table 13 compares the carbon dioxide and carbon monoxide emissions of different consumers for the same system configuration. The values for carbon dioxide and carbon monoxide, the main pollutants, are proportional to the type of consumer.

**Table 13:** Comparison for environmental analysis for low, medium, and large consumer, respectively.

Categories	Carbon dioxide emission (kg/year)	Carbon monoxide emission (kg/year)
Low-consumer	443	0.0217
Medium-consumer	653	0.226
Large-consumer	3,259	0.182

### Policy implications and adoption obstacles

Hybrid energy systems present a unique opportunity for Cameroon's energy sector, yet their successful implementation hinges upon a strategic consideration of their political and socioeconomic ramifications. Key aspects include aggressive energy policies, financing mechanisms, maintenance and training programs, educational campaigns, and regulatory frameworks.

#### Political implications

The political dimension of hybrid energy systems in Cameroon is multifaceted. It is essential to develop and implement energy policies that incentivize the use of renewable energy sources and hybrid systems. Such policies may include the establishment of favorable tariffs, financial incentives, and subsidy programs. Additionally, collaboration between the government, regulatory bodies, and industry stakeholders is crucial to developing customized rules and regulations that facilitate the integration of hybrid systems into the existing energy infrastructure. This collaboration can also lead to the implementation of precise standards and laws that encourage further research and development in the renewable energy sector.

#### Adoption barriers

##### Financial barriers

One of the primary challenges to the widespread adoption of hybrid systems is their high initial costs. These costs can be prohibitive for individuals, businesses, and communities seeking to invest in renewable energy solutions. To overcome this barrier, innovative financing strategies such as credit programs, low-interest loans, and crowdfunding initiatives can be explored. These financing mechanisms can help lower the financial burden and improve access to hybrid systems, particularly for low-income households and small-scale enterprises.

##### Maintenance and training

Ensuring the long-term sustainability of hybrid energy systems requires the availability of skilled technicians and maintenance personnel. Technical know-how is essential for the efficient operation and maintenance of systems that incorporate renewable energy sources, batteries, and other components. Therefore, investing in training programs that equip local employees with the necessary skills for routine maintenance is critical. These training programs can cover a range of topics, including system installation, troubleshooting, and safety protocols. By building local capacity, these programs can contribute to the successful adoption and operation of hybrid systems.

##### Education and awareness

Public awareness and understanding of the benefits of hybrid energy systems play a crucial role in their adoption. Education campaigns can help dispel misconceptions and increase acceptance of renewable energy technologies. These campaigns can target various stakeholders, including policymakers, business owners, and the general public. In addition to highlighting the environmental benefits, such as reduced greenhouse gas emissions and improved air quality, these campaigns can also emphasize the economic advantages of hybrid systems, such as lower energy costs and increased energy security. By fostering a culture of sustainability and energy conservation, education and awareness initiatives can drive demand for hybrid energy solutions.

##### Regulatory obstacles

Regulatory frameworks that are inflexible or outdated can pose significant obstacles to the adoption of hybrid energy systems. In some cases, existing regulations may not adequately address the unique characteristics and requirements of renewable energy technologies. Therefore, collaboration between industry stakeholders and policymakers is essential to identify and address regulatory barriers. This collaboration can lead to the development of updated regulations that accommodate new technologies and promote the integration of renewable energy sources into the energy mix. Additionally, regulatory reforms may be needed to streamline the permitting process for hybrid energy projects and ensure consistency across different jurisdictions.

Through the integration of these considerations into our study, the authors aim to provide a comprehensive understanding of the political, economic, and social factors that influence the adoption of hybrid energy systems in Cameroon. By addressing these factors, the authors hope to contribute to the development of policies and strategies that support the successful implementation and widespread adoption of these systems.

The limitations of this study must be considered when interpreting the findings. Firstly, while the sample size was adequate for our analysis, it was not exhaustive and thus may not capture the entire landscape of renewable energy adoption in the region. Furthermore, the data used in this study was collected from a single location, which limits the generalizability of the findings to other regions or countries. Additionally, the methodology used in this study was based on a systematic literature review and not on direct interviews or surveys with stakeholders, which may have resulted in potential biases or misinterpretations of the findings. Finally, while the authors attempted to account for all relevant variables, there may be other factors that were not considered in this study that could impact the adoption and implementation of renewable energy systems in the region. Overall, while this study provides valuable insights into the current state of renewable energy adoption in Cameroon, further research is needed to fully understand the factors that influence renewable energy adoption and implementation in the region.

## Conclusions and future research directions

Our research meticulously examined the intricacies of a hybrid energy system crafted specifically to cater to the diverse energy demands of Douala, Cameroon. Utilizing advanced simulation tools like the HOMER Pro program, the authors meticulously optimized the hybrid system to attain precise load demand estimates for various consumer segments, thereby facilitating a more nuanced understanding of the region's energy dynamics and requirements. This detailed assessment, coupled with a comprehensive evaluation of diverse pricing models, unequivocally identifies the grid-connected PV/Diesel/Generator system as the most advantageous solution. The system's minimal Net Present Cost (NPC) and Levelized Cost of Electricity (LCOE) underscore its viability, ensuring a reliable and uninterrupted energy supply for consumers across the spectrum of energy usage.

Our research also highlights substantial cost benefits per kilowatt-hour, which is especially pertinent in the context of Cameroon's socio-economic landscape. The authors meticulously calculated updated rates, revealing a significant reduction in costs for low, middle, and high users, further substantiating the economic value proposition of adopting hybrid energy systems.

However, it is essential to underscore the limitations inherent in our study. Variations in input factors like temperature and equipment costs may impact result reliability, warranting a cautious approach when interpreting our findings. Additionally, operational constraints such as maintenance, grid integration, and regulatory compliance pose real-world challenges that cannot be overlooked.

Moving forward, our research sets the stage for enhancing renewable energy integration and developing robust energy management techniques. This work recognizes that overcoming the challenges and obstacles identified in this study is imperative to fully realize the potential of hybrid energy systems. Addressing these issues will be pivotal in mitigating power shortages, fostering sustainable development, and paving the way for a more resilient and sustainable energy future in Cameroon.

Despite the comprehensive nature of our study, several avenues for further exploration exist in the realm of renewable energy systems and their integration within the specific context of Cameroon. Firstly, as technological advancements continue to shape the energy landscape, future research could explore the feasibility and benefits of incorporating emerging renewable energy technologies, such as advanced battery storage systems and more efficient solar panels, into hybrid energy systems.

Secondly, our study predominantly focused on the economic and technical aspects of hybrid energy systems. Future research could delve deeper into the socio-cultural dimensions of renewable energy adoption, examining the perceptions, attitudes, and barriers faced by different societal groups in embracing clean energy technologies.

Thirdly, as Cameroon's energy infrastructure evolves, there is a pressing need for comprehensive energy policies and regulations to guide the deployment and operation of hybrid energy systems. Future research could explore the design and implementation of such policies, taking into account local needs and global best practices.

Lastly, our study primarily focused on a specific region within Cameroon. Future research could expand the scope of analysis to encompass other regions, providing a more holistic understanding of the country's energy landscape and offering tailored solutions to address region-specific challenges.

In conclusion, the future research directions highlighted above underscore the need for continued exploration and innovation in the field of renewable energy systems, with a focus on sustainability, inclusivity, and policy coherence. Such research endeavors will be instrumental in achieving a cleaner, more efficient, and more equitable energy future for Cameroon and beyond.

## Data Availability

The datasets used and/or analysed during the current study available from the corresponding author on reasonable request.
